# Depletion of Foxk transcription factors causes genome-wide transcriptional misregulation and developmental arrest in zebrafish embryos

**DOI:** 10.17912/micropub.biology.000341

**Published:** 2020-12-08

**Authors:** Fan-Suo Geng, Elisa de la Calle-Mustienes, José Luis Gómez-Skarmeta, Ryan Lister, Ozren Bogdanovic

**Affiliations:** 1 Genomics and Epigenetics Division, Garvan Institute of Medical Research, Sydney, New South Wales, 2010, Australia; 2 St Vincent's Clinical School, Faculty of Medicine, University of New South Wales, Sydney, New South Wales, 2010, Australia; 3 Centro Andaluz de Biología del Desarrollo, CSIC/Universidad Pablo de Olavide, Sevilla, Spain; 4 Deceased; 5 Harry Perkins Institute of Medical Research, Perth, 6009, WA, Australia; 6 Australian Research Council Centre of Excellence in Plant Energy Biology, School of Molecular Sciences, The University of Western Australia, Perth, 6009, WA, Australia; 7 School of Biotechnology and Biomolecular Sciences, University of New South Wales, Sydney, New South Wales, 2052, Australia

**Figure 1. Morpholino (MO) knockdown of foxk1/foxk2a/foxk2b and transcriptional profiling of triple foxk morphants:**
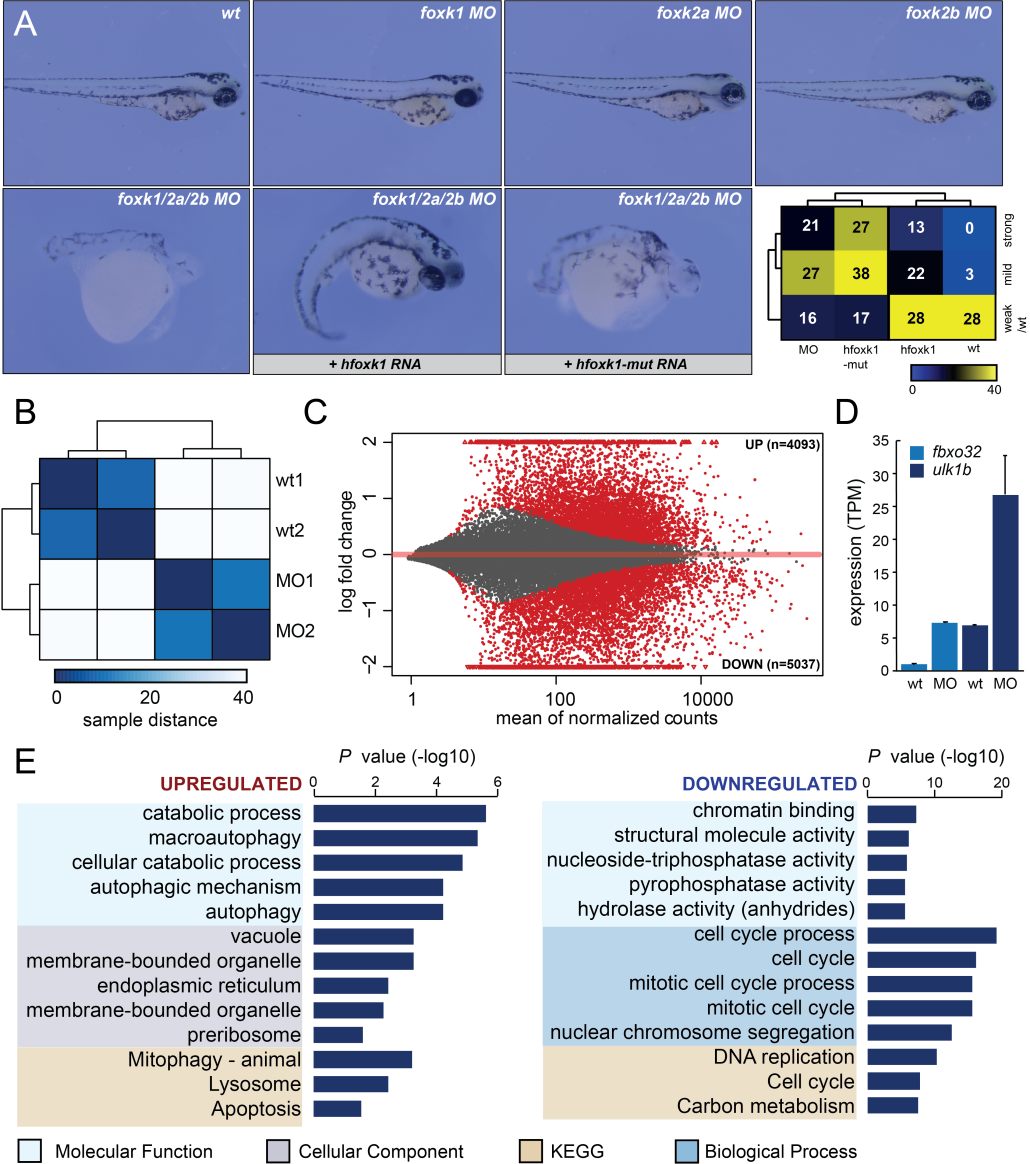
**A)** Upper panels:Zebrafish larvae (72 hpf) injected with morpholinos targeting single *foxk* transcripts. Lower panels: triple (*foxk1/foxk2a/foxk2b*) MO and rescue with full length *(+ hfoxk1* RNA) or mutated *(+ hfoxk1-mut*) human *foxk1* transcripts, at 24hpf. Lower right panel: Hierarchical clustering of embryo numbers corresponding to: triple MO, triple MO with full length *foxk1* transcript, triple MO with mutated *foxk1* transcript, and wild type, divided into phenotype severity groups (weak/wt: no notable developmental delay; mild: minor developmental delay, trunk curvature, pigmentation issues; strong: severe developmental delay, nervous system defects, shortened body axes). **B)** Similarity distance matrix of RNA-seq data (counts) corresponding to wild type (wt) and triple morpholino (MO) conditions. **C)** MA plot displaying upregulated and downregulated genes (red dots) caused by triple Foxk MO. **D)** Expression profiles (TPM) of previously described Foxk targets (*fbxo32*, *ulk1b*) in wt and triple MO conditions. **E)** Gene ontology analysis of the most significantly (n=500) upregulated and downregulated genes in the triple MO condition.

## Description

Foxk proteins are transcription factors from the forkhead box family, implicated in diverse biological processes such as glucose metabolism, inhibition of myogenic differentiation, and repression of autophagy (Bowman *et al.* 2014; Shi *et al.* 2012; Sukonina *et al.* 2019). Recent work has demonstrated that Foxk proteins predominantly act as transcriptional repressors in muscle cells and fibroblasts, where they recruit the Sin3-HDAC complex to silence targeted loci (Bowman *et al.* 2014). However, Foxk proteins can also associate with the DNA demethylase TET1 *in vivo* and *in vitro* (Sun *et al.* 2016), which is suggestive of their role in transcriptional activation and formation of open chromatin. To date, the majority of Foxk studies have been carried out in cultured cells, with limited information available on *in vivo* developmental requirements for Foxk function (Sukonina *et al.* 2019).

To investigate the contribution of Foxk proteins to zebrafish embryonic development, we explored the steady state levels of *foxk* transcripts using the zebrafish developmental expression atlas (https://www.ebi.ac.uk/gxa/experiments/) and found them to be on average highly expressed (10 – 125 TPM) during the first 48 hours of embryogenesis (White *et al.* 2017). We next sought to investigate how the depletion of Foxk proteins affects early embryonic development by employing targeted morpholino (MO) knockdown of three zebrafish Foxk orthologs: Foxk1 (*forkhead box K1*) , Foxk2a (*forkhead box K2a*) , and Foxk2b (*forkhead box K2b*) (**Fig. 1A**). Zebrafish embryos were injected at 1cell stage with MOs targeting either single *foxk* transcripts or all three transcripts together (*foxk1/foxk2a/foxk2b* – triple MO). Whereas the injections of single *foxk* MOs had no notable effect on embryonic development, the triple MO resulted in severe developmental phenotypes including grave nervous system defects and abnormally short bodies in 32% of injected embryos (**Fig. 1A**). We next attempted a rescue assay with full length, wild type human *foxk1* transcript (*hfoxk1*) as well as with a mutant transcript (*hfoxk1-mut*), which contains a point mutation (H355A) that is predicted to disrupt its DNA-binding domain (Freddie *et al.* 2007). The wild type transcript reduced the number of affected embryos, from 75% displaying strong or mild phenotype, to 55%. This effect was not observed when *foxk1* mutant RNA was injected together with the triple MO mixture (**Fig. 1A**).

To assess how the loss of Foxk function affects the embryonic transcriptome, we extracted RNA from 24hpf embryos and subjected it to stranded RNA-seq library preparation and sequencing, generating an average of 40 million reads per sample. Upon mapping, we first generated a distance matrix to obtain insight into similarities between samples (**Fig. 1B**). The data separated by condition, as expected from the experimental design. We next employed differential gene expression analysis (Love *et al.* 2014) to understand the impact of Foxk loss on gene expression. We detected 9,130 differentially expressed genes (4,093 upregulated and 5,037 downregulated in triple MO, p < 0.05), suggestive of significant transcriptional changes caused by the loss of Foxk function (**Fig. 1C**). To validate these RNA-seq results, we searched the literature for previous examples of genes upregulated upon Foxk loss. Notable transcriptional upregulation was observed from two loci (*fbxo32* and *ulk1b*) in the triple MO condition, which were previously reported as Foxk targets in mammalian cells (Bowman *et al.* 2014) (**Fig. 1D)**. To obtain further insight into genes that become misregulated upon Foxkknockdown, we performed gene ontology analysis (Reimand *et al.* 2007) on the most highly affected upregulated and downregulated genes. Genes upregulated in the triple MO displayed significant enrichment in ontologies associated with autophagy and starvation (**Fig. 1E)**,whereas downregulated genes were highly enriched in cell cycle regulators. Foxk1 proteins have previously been implicated in cell cycle control (Grant *et al.* 2012). We also observed downregulation of genes associated with hydrolase activity, in line with the proposed roles of Foxk proteins in autophagy (Bowman *et al.* 2014). It is worth noting, however, that the tissue complexity of 24hpf embryos as well as the strong phenotypes characterised by significant developmental delay, make it challenging to draw precise conclusions regarding the impact of Foxk proteins on transcriptional regulation.

Our comprehensive assessment of Foxk function in zebrafish embryos demonstrates that Foxk proteins display redundant roles during embryogenesis, as evidenced by the lack of phenotype upon single *foxk* MO injections (**Fig. 1A**). The triple MO severely affected early embryogenesis but was nevertheless ameliorated by the injection of a full length human *foxk1* transcript. The transcript coding for mutated Foxk1 that inhibits DNA binding was not able to rescue the triple MO phenotype, suggestive of a requirement for Foxk DNA binding during embryogenesis (**Fig. 1A**). The triple MO resulted in global transcriptional misregulation, including upregulation of autophagy-related genes and downregulation of cell cycle regulators (**Fig. 1C-E**). Overall, zebrafish recapitulates the majority of molecular phenotypes previously associated with Foxk loss *in vitro* and can serve as a useful model system for the further exploration of Foxk function *in vivo*.

## Methods

**Foxk morpholino knockdown and rescue experiments:** Either 9 ng of a single morpholino or 3 ng each for the combination of all three morpholinos (9ng total) was injected into one-cell stage zebrafish embryos. The observed phenotypes were recorded at different time points using a stereoscope (SZX16-DP71, Olympus). Full length and mutant (H355A) human *foxk1* RNA required for rescue experiments were *in vitro* transcribed from pAS2255 and pAS2259 plasmids (Freddie *et al.* 2007), respectively.

**RNA-seq library preparation and data analysis:** RNA-seq libraries were prepared using the TruSeq Illumina stranded RNA-seq kit following manufacturer’s instructions, starting with 500 ng of RNA per sample. The libraries were sequenced on the Illumina HiSeq1500 sequencing platform. RNA-seq reads were trimmed to remove adapter sequences and low-quality nucleotides with Trimmomatic (Bolger *et al.* 2014) and mapped using Kallisto (Bray *et al.* 2016) with the following settings: kallisto quant –single -l 100 -s 20 -t 4. The sequence required for the generation of the reference transcriptome (Danio_rerio.GRCz11) was obtained from ENSEMBL. Differential gene expression analysis was conducted in R with the DEseq2 package (Love *et al.* 2014; Zhu *et al.* 2019). Gene ontology analysis was performed with gProfiler (Reimand *et al.* 2007) on 500 most upregulated and 500 most downregulated genes, sorted by adjusted P values, as per DEseq2 results. RNA sequencing data have been deposited to the ArrayExpress database (https://www.ebi.ac.uk/arrayexpress) under accession number E-MTAB-9777.

**Animal handling:** All animal experiments were conducted following the guidelines established and approved by the local governments and the Institutional Animal Care and Use Committee, (Universidad Pablo de Olavide, Spain), always in accordance with best practices outlined by the European Union.

## Reagents

The sequences of morpholinos targeting each foxk transcript are as follows:

*foxk1* (Ensembl ID: ENSDARG00000037872): CGGTATCATCCCCTAAATCAGCCAT

*foxk2a* (Ensembl ID: ENSDARG00000030583): CCATCTGTACCGCCGCTGACCGGGA

*foxk2b* (Ensembl ID: ENSDARG00000011609): ACGGGCCATCGCTGCCATCTTTATC
